# Supplementary material for: Identification of 55,000 Replicated DNA Methylation QTL
Source: Sci Rep. 2018 Dec 4;8:17605. doi: 10.1038/s41598-018-35871-w (PMC6279736; doi:10.1038/s41598-018-35871-w)
Supplement: Supplementary file 1 — Supplementary Figures and Tables [file 41598_2018_35871_MOESM1_ESM.pdf]

Supplementary Information

Identification of 55,000 Replicated DNA Methylation QTL

Allan F McRae, Riccardo E Marioni, Sonia Shah, Jian Yang, Joseph E. Powell,

Sarah E Harris, Jude Gibson, Anjali K Henders, Lisa Bowdler, Jodie N. Painter, Lee Murphy,

Nicholas G Martin, John M Starr, Naomi R Wray, Ian J Deary, Peter M Visscher,

and Grant W Montgomery

Figure S1: Distance of *cis* mQTL SNP from target CpG site. Most of the significant *cis* mQTL SNP are located within 100Kbp from the target CpG. However, substantial numbers of *cis* mQTL are still observed at 1-2Mbp away from the target CpG site.

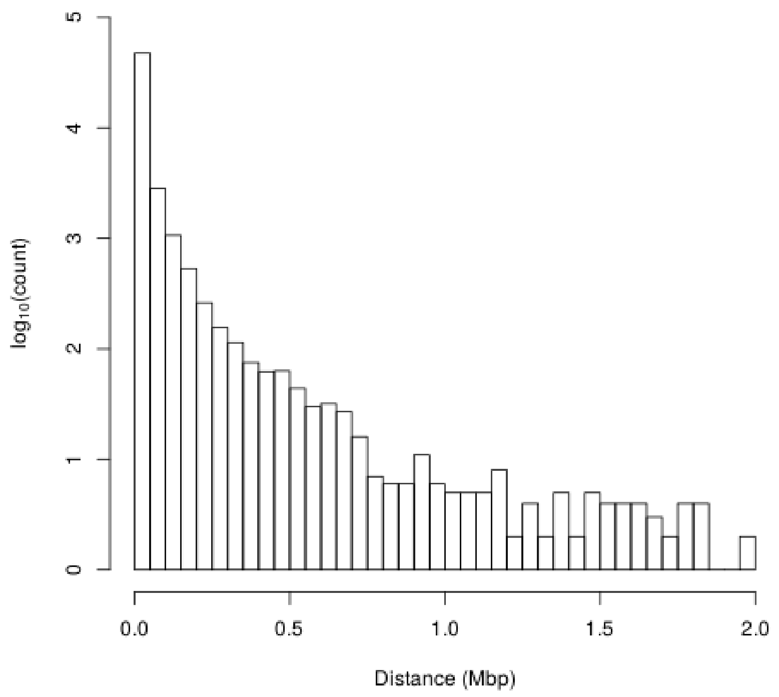

Figure S2: Location of significant mQTL in the BSGS (left) and LBC (right) cohorts. There is a substantial overlap in significant mQTL between the two groups.

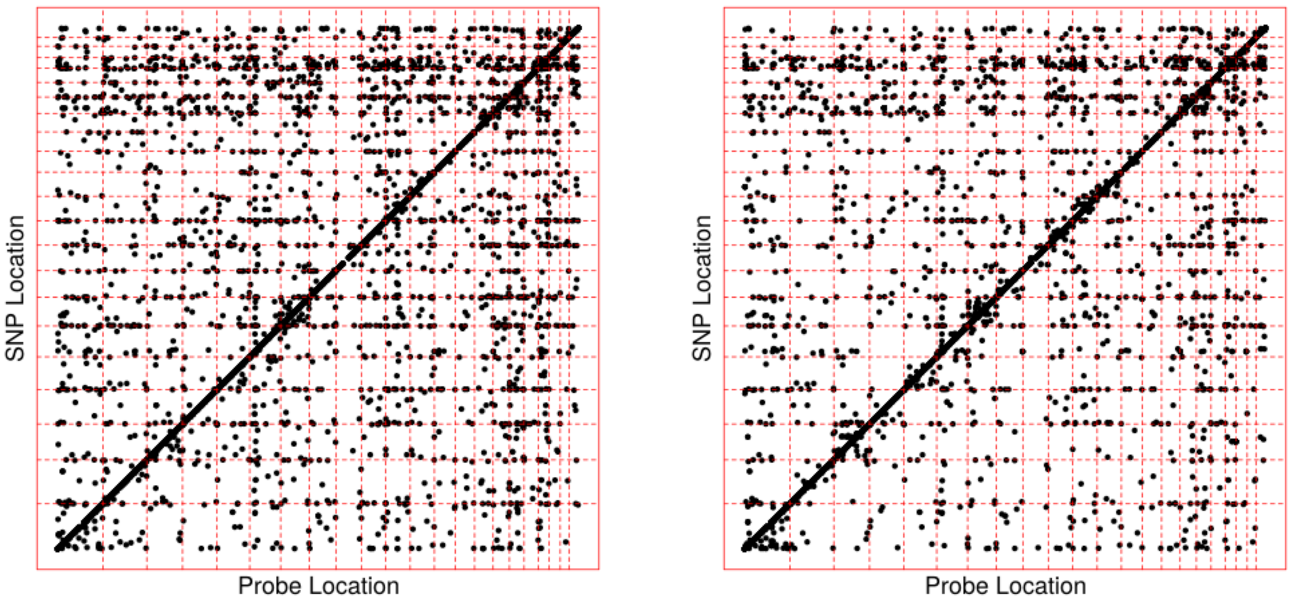

Figure S3: No association between *trans* mQTL SNP and methylation with telomere length in the LBC. Both the SNPs (a) and methylation (b) from *trans* mQTL showed no inflation in their average test statistics for association with telomere length. Black dots are for SNPs/methlation probes genome wide and SNPs/probes from *trans* mQTL are coloured blue.

(a)

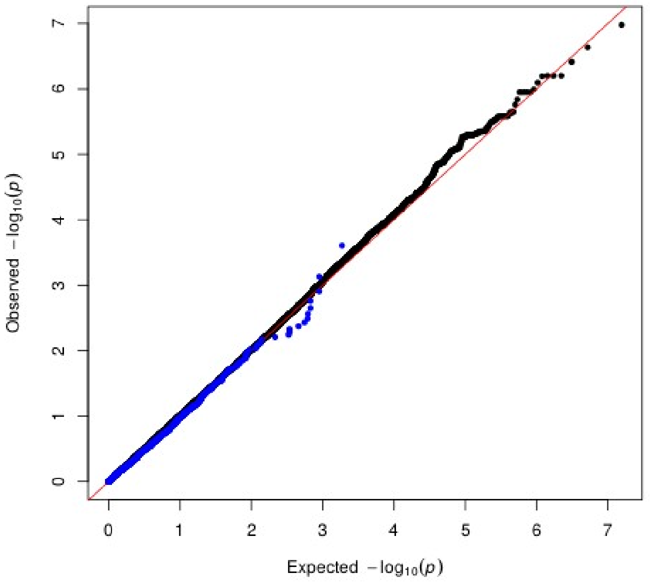

(b)

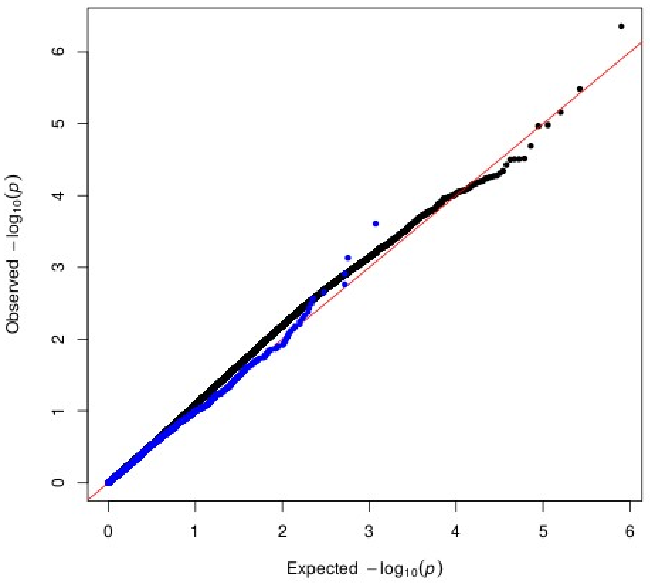

Supplementary Table 1: List of replicated mQTL. Excel spreadsheet containing the list of all 52,916 *cis* and 2,025 *trans* mQTL identified and replicated in the BSGS and LBC cohorts. All genomics positions are given with hg19 locations.

Supplementary Table 2: Correlation between the absolute value of the mQTL effect size and the absolute value of the SNP effect size or log-odds ratio for a number of complex traits and disease. Significant correlations are observed for all traits tested but this is driven purely by the relationship between average effect size and allele frequency.

**Trait**	**Raw Effect Sizes**		**Standardized Effect Sizes**	
	**Correlation**	**P-value**	**Correlation**	**P-value**
Height	0.048	2×10^{-8}	-0.004	0.69
BMI	0.072	$< 2 \times 10^{-16}$	0.002	0.42
Schizophrenia	0.082	$< 2 \times 10^{-16}$	0.004	0.26
Ulcerative colitis*	0.045	8×10^{-6}	-0.024	0.99
Crohn's Disease*	-0.010	0.82	0.001	0.45
Coronary Artery Disease	0.050	8×10^{-9}	-0.020	0.99
Type 2 Diabetes	0.100	$< 2 \times 10^{-16}$	0.004	0.34
Rheumatoid Arthritis*	0.092	$< 2 \times 10^{-16}$	-0.007	0.87
Educational Attainment	0.050	1×10^{-8}	0.004	0.34

* Excluding the HLA region of chromosome 6
